# Systematic Assessment of Gambling Type Involvement: Reliability and Validity of the Gambling Disorder Identification Test (GDIT)

**DOI:** 10.1007/s10899-024-10345-z

**Published:** 2024-08-02

**Authors:** Håkan Wall, Peter Wennberg, Per Binde, Olof Molander

**Affiliations:** 1 Department of Clinical Neuroscience, Centre for Psychiatry Research, Karolinska Institutet, Norra Stationsgatan 69, Plan 7, Solna, Stockholm, 113 64 Sweden; 2 Stockholm Region Health Services, Stockholm, Sweden; 3 Department of Public Health Sciences, Stockholm University, Stockholm, Sweden; 4 Department of Global Public Health, Karolinska Institutet, Solna, Sweden; 5 Department of Psychology, Inland Norway University of Applied Sciences, Rena, Norway

**Keywords:** Gambling type involvement, Psychometric, Assessment, Problem gambling, The gambling disorder identification test

## Abstract

Gambling type involvement, both in terms of participation (engagement in specific gambling types) and diversity (how many gambling types an individual engages in), is a key feature to address in gambling self-report measures, but such systematic measurement procedures are scarce. The aim of this study was to test the psychometric performance of the gambling type assessment in the recently developed Gambling Disorder Identification Test (GDIT), in terms of test-retest reliability, convergent validity, and patterns of gambling diversity, among help-seeking and general population gambling samples (total *n* = 603). Overall, online gambling was more commonly reported as problematic than land-based gambling. Retest reliability varied for specific gambling types (ICC range 0.32–0.64, r_tet_ range 0.66–0.85). In terms of gambling participation, online gambling showed stronger correlations with GDIT total score (i.e., symptom severity) than land-based gambling, where Slots showed the strongest correlation (*r* = 0.52), followed by Casino table games (*r* = 0.25), Sports and Horse betting (*r* = 0.16 and *r* = 0.14, respectively), and Poker (*r* = 0.14). Lotteries showed no correlation with GDIT total score (*r*=-0,01). For Slots gambling, all gambling diversity levels (including Slots as a single gambling type) were on average associated with the highest diagnostic severity level (GDIT total score > 30; severe gambling disorder). Finally, explorative configural frequency analysis identified typical and antitypical gambling diversity patterns. The result from the current study corroborates findings that engagement in specific gambling types matter, and that such features should be included in gambling measurement. We conclude that the GDIT is a reliable and valid measure for systematic assessment of gambling type involvement. The GDIT can be used to assess gambling participation and diversity, as part of a broad measurement setup for problem gambling and gambling disorder.

## Introduction

Gambling comes in many forms or types, which have their specific combinations of design features, such as speed of play, continuity of play, and the intensity of sensory stimuli (Allami et al., 2021; Parke et al., 2016). The social and cultural contexts of types of gambling also varies (Matilainen & Raento, 2014), as do the perceived skill and luck influencing gambling outcome (Browne et al., 2015; Lopez-Gonzalez et al., 2021). These features and contexts are all potential risk or protective factors for problem gambling (PG) and the DSM-5 diagnostic criteria of gambling disorder (GD; American Psychiatric Association, 2013). Gambling involvement is a broad term for engagement in gambling types (LaPlante et al., 2014), including both gambling *participation* and *diversity* (see below).

The term gambling participation describes engagement in specific gambling types. Research has consistently found that some gambling types have a stronger association with PG than other (Binde, 2017; Mazar et al., 2020). Electronic gambling machines (EGMs) have typically the strongest association (Browne et al., 2023; Wall et al., 2021) while traditional lotteries have in general a weak association. This can be explained by EGMs offering high speed and continuity, as well as conspicuous sensory stimuli crafted to stimulate the player´s willingness to gamble (Schüll, 2012). Lotteries, on the other hand, are slow, discontinuous, and offer few sensory stimuli. Other gambling types have associations with PG in between, the precise order varying between jurisdictions and time periods (Binde, 2017). There are two modes of access to gambling. Physically – such as in a betting shop or a brick-and-mortar casino – or online via the Internet. Today, online gambling provides easy and immediate access to gambling. Online casinos and EGMs have made it possible for numerous people, who earlier had no close physical proximity to places offering such games, to engage in these gambling types whenever they like. This initially increases the risk for these people to develop PG (Abbott, 2020) and more generally to sustain ongoing PG.

The term gambling *diversity* describes how many gambling types an individual engages in. PG correlates with the number of gambling types regularly engaged in (weekly and monthly), and individuals with PG are more likely than people without to diversify their gambling (Binde et al., 2017; Wall et al., 2021). It has been claimed that gambling diversity is such an important PG factor that, when statistically controlled for, it overrides the PG-association of any specific gambling type (LaPlante et al., 2011, 2013). However, this assertion has been shown to rest on questionable use of statistics (Binde et al., 2017). In the Swedish context, it has been shown that, in general, gambling diversity correlates positively with PG, but that the relationship is complex across the forms of gambling (Binde et al., 2017; Wall et al., 2021). EGMs have a strong association with PG regardless of the degree of gambling diversity (Binde et al., 2017; Wall et al., 2021). A substantial number of people with PG were in the two studies by Binde et al. (2017) and Wall et al. (2021) found to regularly participate in EGM gambling only. One of the studies found that regular horse betting appears to be a PG-protective factor (Binde et al., 2017), presumably because of the sociocultural emphasis on betting skillfully and under strong self-control among dedicated horse bettors (Binde, 2011).

Gambling type involvement (both in terms of participation and diversity) is thus a key feature to address in gambling self-report measures. In 2006, an agreement was formed among gambling researchers on a minimum set of features to measure when conducting gambling research (Walker et al., 2006), which included recommendations on gambling type involvement. The agreement, commonly referred to as the Banff consensus, acknowledged that individuals could engage in multiple gambling types (i.e., diversity), and further emphasized that it is only involvement in problematic gambling types that should be reported. At the same time, universal definitions on gambling type involvement have been lacking (Williams et al., 2017). Apart from the Gambling Participation Inventory (GPI; Williams et al., 2017), which is a comprehensive self-report measure specifically developed to assess gambling involvement, systematic measurement procedures are scarce. Items targeting gambling type involvement have seldom been included as formal parts of existing gambling measures (see Molander et al., 2019, for an analysis).

**Fig. 1:**
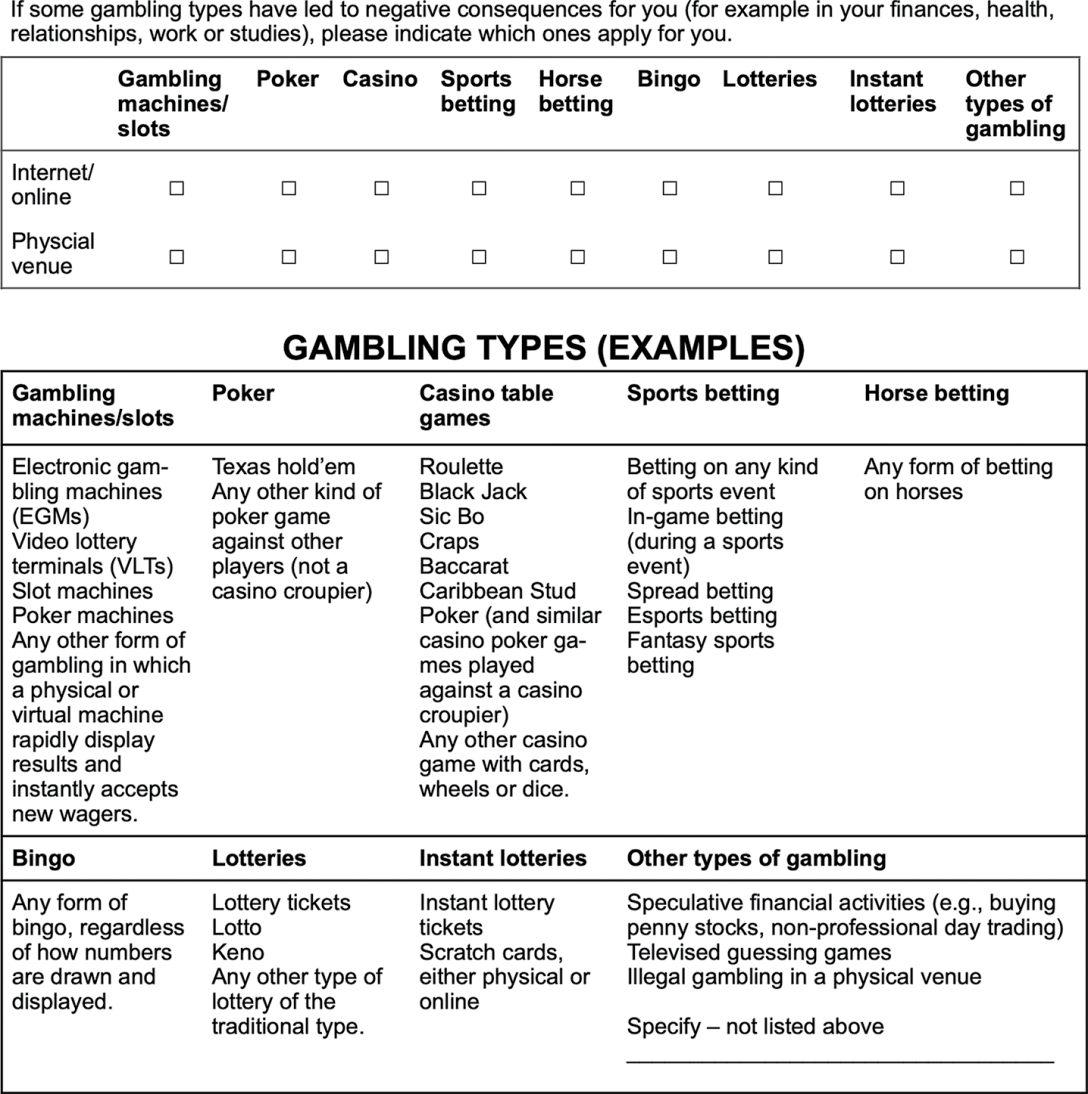
Gambling type assessment in the GDIT

The Gambling Disorder Identification Test (GDIT) was recently developed as a comprehensive self-report measure to assess PG and GD, using the same measurement format as the Alcohol Use Disorders Identification Test (AUDIT; Saunders et al., 1993) and the Drug Use Disorders Identification Test (DUDIT; Berman et al., 2004). The GDIT development process (Molander et al., 2021) included categorization of problematic gambling type participation: Slots (including EGMs), Poker, Casino table games, Sports betting, Horse betting, Bingo, Lotteries, Instant lotteries, and Other gambling types; accessed online and/or at venues/land-based) - with examples and definitions of gambling types provided in a table, similar to the List of Drugs in the DUDIT. See Fig. 1 for the gambling type assessment of the GDIT.

The general psychometric properties and performance of GDIT have been explored in a previous publication (Molander et al., 2021). This article presents results from a study focused on the gambling type classification and measurement of the GDIT. The results are relevant not only in the psychometric context, but also add to the knowledge of the association between PG/GD and specific gambling types, and between PG/GD and gambling diversity.

The aim of this study is to test the psychometric performance of GDIT’s gambling type assessment in terms of test-retest reliability, convergent validity, and patterns of gambling diversity. Regarding convergent validity, we predicted, based on previous research on gambling participation and diversity, that: (1) Online gambling will show stronger correlations with GDIT total score than land-based gambling, (2) Slot gambling will show the strongest correlation with GDIT total score among the gambling types, and (3) multiple gambling type engagement (diversity) will show stronger associations with GDIT total score than engagement in a single gambling type, with the exception of Slot/EGM gambling.

## Methods

### Participants and Procedure

The study analyzed data from a previous psychometric study which evaluated the GDIT (see Molander et al., 2021). In the psychometric study gamblers (total *n* = 603) from treatment- and help-seeking contexts, self-help groups and the general population, were recruited. Participants completed self-report measures in an online survey, and a (6 to 16 days) GDIT re-test measure. The psychometric study was approved by the Regional Ethics Board of Stockholm, Sweden (ref. no. 2017/1479-31), and all participants provided informed consent for study participation and publication of results, including the results published in this study. In the current study, analyses were performed on two gambling cohorts: general population, and gamblers from self-help groups, support- and treatment-seeking samples, collapsed into a help-seeking cohort. See Table 1 for participant characteristics.

**Table 1:** Participant characteristics across samples

	Gambling cohort			Gender^a^			Total
Characteristics	General population *n* = 292	Help-seeking *n* = 311		Men *n* = 444	Women *n* = 152		Total N = 603
Age, M (Sd)	29.5 (10.5)			31.6 (11.8)	37.5 (12.1)		33.0 (12.1)
Source of income, %							
Employed	60	69		64	66		64
Studies	30	12		23	13		21
Other	10	20		13	21		15
Highest level of education, %^b^							
University	46	25		34	36		35
High school	45	59		53	51		52
Junior high school	8	13		10	12		10
Relationship status, %							
Cohabiting	70	64		68	62		67
Parental status, %							
Having children	30	51		36	57		41
Gambling characteristics							
Gambling debts, %	8	63		32	50		36
Access mode, %							
Online gambling	46	81		63	68		64
Gambling at venues	24	25		26	21		25
Gambling participation (%)							
Slots, online	17	55		31	55		36
Slots, venue	9	10		9	9		9
Poker, online	12	14		17	3		13
Poker, venue	4	4		5	1		4
Casino table games, online	10	25		19	13		18
Casino table games, venue	4	5		5	2		5
Sports betting, online	14	20		22	4		17
Sports betting, venue	4	10		10	0		7
Horse betting, online	6	10		9	5		8
Horse betting, venue	4	5		6	2		5
Bingo, online	2	4		2	5		3
Bingo, venue	1	2		1	1		1
Lotteries, online	4	7		6	5		6
Lotteries, venue	2	3		3	3		3
Instant lotteries, online	3	6		4	5		4
Instant lotteries, venue	4	4		3	6		4
Other, online	5	5		6	3		5
Other, venue	5	4		5	3		4
Gambling diversity							
*n* gambling types, M (Sd)	1.03 (1.33)	1.74 (1.42)		1.47 (1.51)	1.2 (1.12)		1.39 (1.42)
No gambling type, %	47	16		30	28		29
One gambling type, %	16	38		23	36		26
Two gambling types, %	17	25		19	16		19
Three gambling types, %	8	8		9	12		10
Four gambling types, %	4	5		6	1		5
Five or more gambling types, %	8	9		12	6		10

*Note* Participants were able to report several problematic gambling types

^a^Seven participants reported that they could/would not state their gender

^b^15 participants did not report level of education

### Measures

#### The GDIT

The GDIT (Molander et al., 2021; www.gditscale.com), is a recently developed self-report measure to assess GD and PG. The GDIT consists of 14 items with frequency-based response alternatives within in three domains: Gambling behavior GDIT_1 − 3_ (scored 0–6), PG/GD symptoms GDIT_4 − 10_ (scored 0–4), and negative consequences GDIT_11 − 14_ (scored 0, 2 or 4). The maximum GDIT total score is 62. A GDIT total cut-off score of ≥ 20 indicate a diagnosis of GD, and total cut-off scores of 20, 25, and ≥ 30, mild, moderate, and severe GD symptom severity, respectively. GDIT total scores of ≥ 15 indicate problem gambling, while GDIT total scores < 15 corresponds to recreational gambling.

Engagement in perceived problematic gambling types (i.e., Slots [including EGMs], Poker, Casino table games, Sports betting, Horse betting, Bingo, Lotteries, Instant lotteries, and Other gambling types) are measured via a check-box item grid in the GDIT, where access mode for each gambling type is specified, that is, gambling online or at venues (land-based). The following item instruction is provided: “If some gambling types have led to negative consequences for you (for example in your finances, health, relationships, work or studies), please indicate which ones apply for you.” In addition, examples and definitions of the gambling types are included in a table in the GDIT, similar to the List of Drugs in the DUDIT.

### Statistical Analysis and Data Preparation

An a priori statistical plan was published via aspredicted.org (https://aspredicted.org/q99dg.pdf), and OSF Registries (DOI: 10.17605/OSF.IO/8CJ9Z). When applicable, outcomes were estimated on the total study sample, by gender and by gambling cohort.

Reliability and validity measures were estimated on collapsed dichotomized variables (1, 0), for access mode (gambling types collapsed), and specific gambling types (access modes collapsed). Convergent validity between gambling types and symptom severity (GDIT total score), was estimated using the Pearson correlation coefficient (r). Retest reliability was estimated using the Intraclass correlation coefficient (ICC), and the Tetrachoric correlation coefficient (*r*_tet_), albeit the latter was not included in the original analysis plan.

When testing the effect of increased gambling diversity, GDIT total score was analyzed as count data. Quasi-Poisson regression models were used to prevent an underestimation of the standard errors. To get large enough groups the 17 gambling types in the GDIT were collapsed into four aggregated gambling categories. Online and land-based Slots were collapsed into the aggregated category “Slots”. Online and land-based Poker and Casino table games, were collapsed into the category “Casino games”. Online and land-based Sports and Horse betting were categorized as “Sports betting”. Online and land-based Lotteries, Instant lotteries, and online and land-based bingo were categorized as “Lotteries”. The gambling type Other was excluded from the regression analyses. For each of these four aggregated gambling categories, we tested how the GDIT total score changed by adding other gambling categories in a stepwise order. We started by calculating the GDIT total score for gambling on a certain gambling category and then added other gambling categories incrementally - in step1: a specific gambling category, Step 2: the specific gambling category and one other gambling category (any gambling category), Step 3: a specific gambling category and two or more other gambling categories. To evaluate the effect of increased diversity, we compared GDIT total score for each increment to the reference level (only gambling on a specific gambling category).

Finally, patterns of gambling were explored using Configural Frequency Analysis (CFA; e.g., von Eye & Wood, 1996), which is a statistical procedure to identify patterns that occur more or less frequently than could be expected by chance. In a CFA analysis, observed configurations that occur significantly more often than expected by chance are labeled “types” and observed configurations that occur statistically less often than expected by chance are labeled “antitypes”. To avoid the problem of mass significance, the p-levels were adjusted with Bonferroni’s correction for multiple comparisons. In the CFA analyzes the same gambling type classification as when testing the effect of increased gambling diversity was used.

## Results

### Gambling Participation and Diversity

Overall, online gambling was more commonly reported as problematic than land-based gambling. In the total sample, 36% reported problematic engagement in online Slots, 18% in Casino table games online, 17% in online Sports betting, and 13% in online Poker. About one third of the total sample did not report any problematic gambling type, one in four reported one gambling type, a third reported two to four types, and 10% reported five or more problematic gambling types. See Table 1 for additional descriptives across study samples.

### Test-Retest Reliability

In the total sample, the retest reliability was higher for online gambling (ICC = 0.63, *r*_tet_=0.84), compared to land-based gambling (ICC = 0.45, *r*_tet_=0.68). Retest reliability varied for specific gambling types (ICC range 0.32–0.64, *r*_tet_ range 0.66–0.85), were Slots, Casino table games, Sports betting, Horse betting, and Other gambling types showed higher estimates, and Bingo and Lotteries indicated lower estimates (see Table 2 for retest reliability across study samples). The retest reliability of gambling diversity (*n* gambling types) in the total sample was ICC = 0.62.

**Table 2:** Retest reliability across samples (*n* = 503)

	Gambling cohort			Gender_a_			Total
Test–retest^a^ (6 to 16 days)	General population *n* = 262	Help-seeking *n* = 241		Men *n* = 374	Women *n* = 123		Total *n* = 503
Access mode, ICC | *r*_tet_							
Online gambling	0.57 | 0.79	0.59 | 0.83		0.59 | 0.81	0.73 | 0.91		0.63 | 0.84
Gambling at venues	0.47 | 0.70	0.44 | 0.67		0.41 | 0.63	0.59 | 0.83		0.45 | 0.68
Gambling participation, ICC | *r*_tet_							
Slots	0.51 | 0.76	0.64 | 0.84		0.56 | 0.78	0.78 | 0.94		0.64 | 0.85
Poker	0.56 | 0.82	0.46 | 0.73		0.52 | 0.77	0.19 | 0.52		0.51 | 0.78
Casino table games	0.56 | 0.81	0.58 | 0.81		0.58 | 0.82	0.55 | 0.82		0.58 | 0.82
Sports betting	0.49 | 0.76	0.64 | 0.87		0.57 | 0.81	0.43 | 0.78		0.57 | 0.82
Horse betting	0.52 | 0.82	0.62 | 0.87		0.55 | 0.83	0.73 | 0.96		0.58 | 0.85
Bingo	0.12 | 0.39	0.47 | 0.81		0.32 | 0.68	0.31 | 0.65		0.32 | 0.67
Lotteries	0.25 | 0.54	0.46 | 0.78		0.29 | 0.58	0.56 | 0.85		0.35 | 0.66
Instant lotteries	0.41 | 0.75	0.46 | 0.76		0.35 | 0.67	0.63 | 0.89		0.43 | 0.74
Other	0.54 | 0.84	0.52 | 0.84		0.54 | 0.84	0.49 | 0.86		0.53 | 0.84
Gambling diversity, ICC							
*n* gambling types	0.55	0.66		0.62	0.60		0.62

*Note* The table shows test-retest outcomes for participants in the study who completed a second GDIT test (*n* = 503)

GDIT = The Gambling Disorder Identification Test (Molander et al., 2019)

ICC = Intraclass correlation coefficient

*r*_tet_ = Tetrachoric correlation coefficient

^a^Six participants who completed the GDIT re-test reported that they could/would not state their gender

### Convergent Validity

In line with our first prediction, online gambling showed stronger correlations with GDIT total score (i.e., symptom severity) than land-based gambling. For instance, in the total sample the symptom severity correlations with online and venue gambling were *r* = 0.51 compared to *r* = 0.13, respectively. As stated in our second prediction, Slots showed the strongest correlation with GDIT total score (*r* = 0.52), followed by Casino table games (*r* = 0.25), Sports and Horse betting (*r* = 0.16 and *r* = 0.14, respectively), and Poker (*r* = 0.14). Lotteries had no correlation with GDIT total score (*r*=-0,01). The correlation between gambling diversity (*n* gambling types) and GDIT total score was *r* = 0.40 in the total sample. See Table 3 for validity estimates across study samples.

**Table 3:** Convergent validity, correlations between gambling types and symptom severity, across samples

	Gambling cohort			Gender^a^			Total
Symptom severity (GDIT total score)	General population *n* = 292	Help-seeking *n* = 311		Men *n* = 444	Women *n* = 152		Total *N* = 603
Access mode, *r*							
Online gambling	0.39	0.46		0.45	0.66		0.51
Gambling at venues	0.20	0.12		0.16	0.09		0.13
Gambling participation, *r*							
Slots	0.41	0.39		0.43	0.65		0.52
Poker	0.29	0.09		0.22	0.03		0.14
Casino table games	0.24	0.15		0.33	0.13		0.25
Sports betting	0.13	0.13		0.24	0.11		0.16
Horse betting	0.11	0.14		0.18	0.09		0.14
Bingo	-0.02	0.05		0.02	0.06		0.05
Lotteries	0.01	-0.06		0.00	-0.03		-0.01
Instant lotteries	0.00	0.06		0.07	0.00		0.06
Other	0.04	0.07		0.07	0.00		0.04
Gambling diversity, *r*							
*n* gambling types	0.39	0.30		0.44	0.39		0.40

*Note r* = The Pearson correlation coefficient

GDIT = The Gambling Disorder Identification Test (Molander et al., 2019)

^a^Seven participants reported that they could/would not state their gender

### Gambling Diversity

**Fig. 2:**
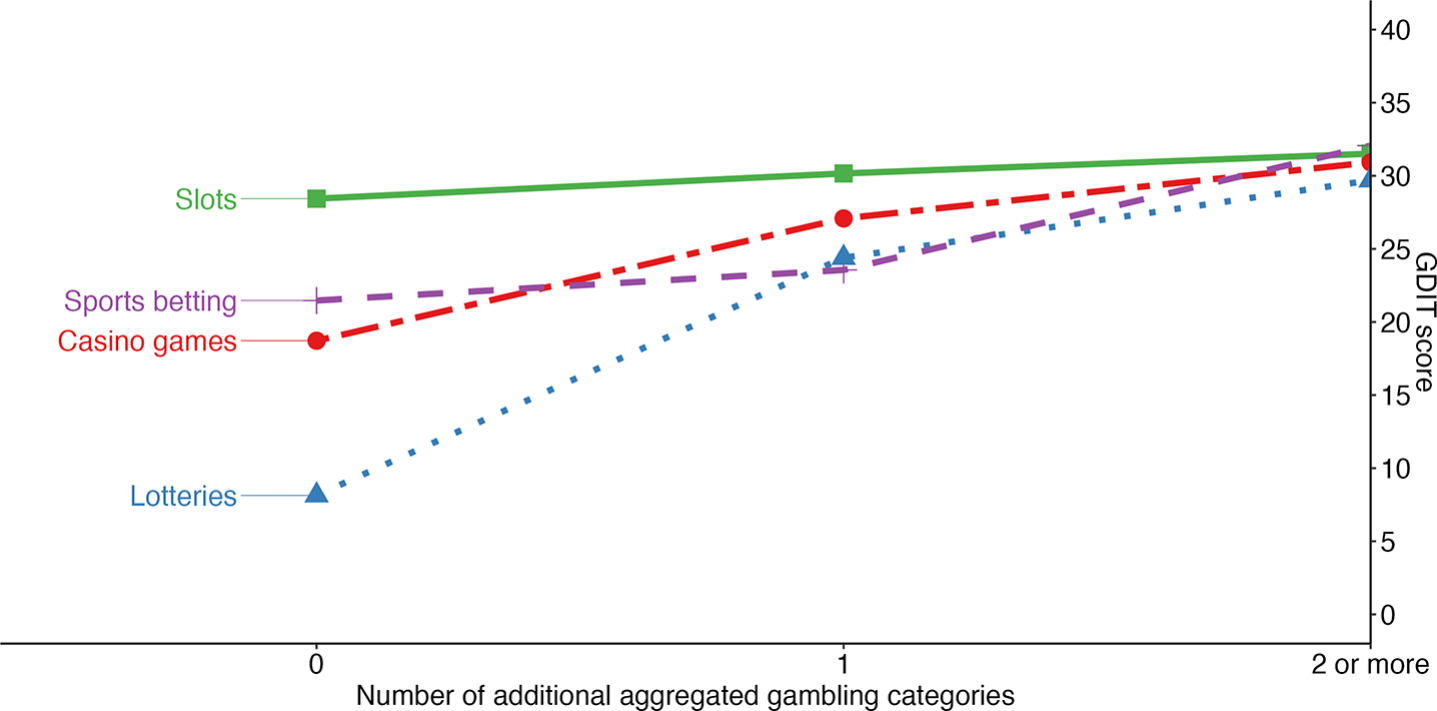
Effect on GDIT total score of increased gambling diversity for four different gambling categories. See Table 4, for specific estimates. Note Slots = slots online and land-based slots. Casino games = poker online, land-based poker, casino games online and land-based casino games. Sports betting = sports betting online, land-based sports betting, horse betting online and land-based horse betting. Lotteries = lotteries online, land-based lotteries, instant lotteries, scratch tickets online, land-based scratch tickets, bingo online, and land-based bingo

In line with our third prediction, we found no significant effect on GDIT total score of increased gambling diversity for Slots. For the other categories, we found that for Sports betting and Casino games, two or more additional gambling categories had to be added to see an effect on increased GDIT score, whereas for Lotteries the GDIT total score increased dramatically (16.3 points, *p* < 0.001) when adding one extra gambling category. See Fig. 2; Table 4 for effects of increased gambling diversity for each gambling category.

**Table 4:** GDIT total score by increased gambling diversity in four different aggregated gambling categories

Number of other gambling categories, mean GDIT total [95% CI]			
	0	1	≥ 2
Slots	28.4 [26.0,31.0]	30.2 [26.1,34.8]	31.5 [26.2,37.7]
Casino games	18.7 [15.0,23.0]	27.1^**^ [21.1,35.0]	30.9^***^ [23.6,40.8]
Sports betting	21.5 [19.9.25.5]	23.6 [18.0,30.8]	32.1^**^ [24.4,42.0]
Lotteries	8.1 [5.2,12.0]	24.4^***^ [15.5,40.0]	29.7^***^ [19.2,48.0]

*Note* * *p* ≤ 0.05, ** *p* ≤ 0.001, *** *p* ≤ 0.0001

The table presents Quasi-Poisson regression GDIT effects of increased diversity for four different gambling categories. See also Fig. 2

GDIT = The Gambling Disorder Identification Test (Molander et al., 2019)

### Patterns of Gambling Diversity

The CFA pattern that included all categories of gambling was typical, i.e., that occurred more often than expected by chance. Conversely, two CFA patterns were antitypical: gambling on Casino games only with no other gambling categories, and Slots and Sports betting but no other gambling categories.

## Discussion

The aim of this study was to test the psychometric performance of the GDIT’s gambling type assessment in terms of test-retest reliability, convergent validity, and patterns of gambling diversity.

The test-retest reliability was moderate to good across access modes (online or land-based), specific gambling types, and sub-samples. This indicates a stability in gambling type involvement, and the GDIT reliably captures this aspect.

Convergent validity was defined and tested via a set of research-informed predictions, which were confirmed by the results. Regarding gambling participation, slots/EGMs showed a particularly strong association with PG/GD and online gambling was more closely associated with PG/GD than land-based gambling, which is in line with previous research (see the Introduction). Increased gambling diversity was also associated with PG/GD, but not for slots/EGM gambling, which was about equally harmful across all diversity levels (see Fig. 2). Most research on gambling diversity has been conducted in relation to PG, and less is known of the diagnostic DSM-5 criteria (Molander et al., 2021). An advantage of the GDIT is that it includes scoring of GD, including severity levels. An important study finding is thus that for slot gambling, all diversity levels (including slots as a single gambling type) were on average associated with being on the highest diagnostic severity level (GDIT > 30; severe GD; American Psychiatric Association, 2013) (see Fig. 2). As for lotteries, they had a markedly weak association with PG/GD by themselves, but those who participate in lotteries as well as other categories of gambling ran a risk for PG/GD which was almost as high as for those who participated only in slots. Sports betting and casino games had associations with PG/GD which were in between those of slots and lotteries. This is consistent with previous research (see the Introduction) as well as data from Swedish population studies on gambling (e.g., The Public Health Agency of Sweden, 2021, Wall et al., 2021, Gooding & Williams, 2023).

Regarding patterns of gambling diversity, three CFA patterns were statistically significant; two of these were “antitypes” that was less common than predicted and one pattern a “type” that was more common than predicted (Table 5). We speculate that the first antitypical pattern – participating in slots and sports betting, but no other gambling categories – reflect the dichotomous relationship between slots and sports betting (Balodis et al., 2014). It has been found that, in general, females prefer slots to sports betting (Gausset & Jansbøl, 2009), that some gamblers prefer luck games rather than “skill” games (Huggett et al., 2021), and that slots are associated with “escape” motives (Abarbanel, 2014), while sports betting is more associated with a desire to win money (Ibid.) as well as sensation seeking (Bonnaire et al., 2006). The second antitypical pattern is participating in casino games but no other games. This might reflect that people who visit online casinos are very likely to play also slots while they are logged in. The third “type” pattern is participating in all four categories of gambling. This pattern is probably more common than others because it refers to people who have a taste for gambling, or an addiction to it, and gamble indiscriminately on whatever gambling types that comes their way (Studer et al., 2016).

**Table 5:** Distributions of patterns of gambling diversity

Gambling type categories					Frequency (*n*)			
Slots	Casino games	Sports betting	Lotteries		Observed	Expected	*P* ^a^	Significant type/antitype
No	No	No	No		211	191	0.069	-
Yes	No	No	No		125	120	0.329	-
No	No	No	Yes		22	25	0.254	-
Yes	No	No	Yes		12	16	0.160	-
No	No	Yes	No		62	56	0.202	-
Yes	No	Yes	No		15	35	0.000	Antitype
No	No	Yes	Yes		7	7	0.439	-
Yes	No	Yes	Yes		1	5	0.045	-
No	Yes	No	No		39	63	0.001	Antitype
Yes	Yes	No	No		47	40	0.120	-
No	Yes	No	Yes		4	8	0.066	-
Yes	Yes	No	Yes		8	5	0.117	-
No	Yes	Yes	No		22	18	0.200	-
Yes	Yes	Yes	No		13	12	0.340	-
No	Yes	Yes	Yes		4	2	0.160	-
Yes	Yes	Yes	Yes		13	1	0.000	Type

*Note* Slots = Slots = slots online and land-based slots

Casino games = poker online, land-based poker, casino games online and land-based casino games

Sports betting = sports betting online, land-based sports betting, horse betting online and land-based horse betting

Lotteries = lotteries online, land-based lotteries, instant lotteries, scratch tickets online, land-based scratch tickets, bingo online, and land-based bingo

^a^After Bonferroni’s correction for multiple comparisons

Although the study was conducted in a Swedish setting, it can be argued that the results have bearing in other jurisdictions as well, at least in the European context, where most gambling markets resemble one another (licensing markets) with regard to gambling products and regulation. However, the association between particular gambling types might vary in strength between jurisdictions, depending on gambling cultures and the structure of the gambling market (Williams et al., 2012). For example, land-based EGMs in a Swedish context are few in number, have a loss limit, and low bet size compared to for instance the Australian context, this may limit this study’s generalizability to some extent. Finally, we note that an important task when screening for PG/GD is to identify new gambling types (or similar phenomena/products), in order to understand what the individuals currently are struggling with. Instead of just offering a tick box named “other gambling types” with an open-ended text field, examples (which can be culturally adapted) of gambling like products should be specifically mentioned, such as “speculating in crypto currencies”, “crypto currency gambling”, “speculating on the stock market”, “skin betting” or “gambling like features in gaming”. This would expand our understanding of emerging trends.

This was a psychometric study, but it also contributed to the general knowledge about the PG/GD association with gambling participation and diversity. Further strengths were that gamblers from both low and high severity populations were included in the sample, and that convergent validity was tested using a set of predefined research-informed predictions. Regarding limitations, the study used a relatively small sample, which had to be addressed by aggregating gambling types into collapsed categories for some analyses. Future research could corroborate findings among international samples, develop a taxonomy of gambling types based on their association to PG/GD severity, or investigate the predictive value of participation in specific gambling types for PG/GD screening purposes. Last, we emphasize that measurement of gambling involvement needs to be open to adaptations, as this is a dynamic field where different gambling types continuously are being introduced or disappearing.

## Conclusions

The result from the current study corroborates findings that engagement in specific gambling types matter, and that such features should be included in gambling measurement. Overall, we conclude that the GDIT is a reliable and valid measure for systematic assessment of gambling type involvement. The GDIT can be used to assess gambling participation and diversity, as part of a broad measurement PG/GD setup. For in-debt comprehensive measurement of gambling type involvement, we recommend using the GPI (Williams et al., 2017). The study further corroborated findings that certain gambling types are more related to PG/GD severity than others, and we urge that these should be targeted in clinical and public-health settings, for instance by specifying problematic gambling types in low-risk gambling guidelines.

## Data Availability

The data that support the findings of this study are available from the corresponding author upon request.
